# Assembly, stability, and dynamics of the infant gut microbiome are linked to bacterial strains and functions in mother’s milk

**DOI:** 10.1101/2024.01.28.577594

**Published:** 2024-01-28

**Authors:** Mattea Allert, Pamela Ferretti, Kelsey E. Johnson, Timothy Heisel, Sara Gonia, Dan Knights, David A. Fields, Frank W. Albert, Ellen W. Demerath, Cheryl A. Gale, Ran Blekhman

**Affiliations:** 1 Department of Genetics, Cell Biology, and Development, University of Minnesota, Minneapolis, MN, USA; 2 Section of Genetic Medicine, Department of Medicine, University of Chicago, Chicago, IL, USA; 3 Department of Pediatrics, University of Minnesota, Minneapolis, MN, USA; 4 Department of Computer Science and Engineering, University of Minnesota, Minneapolis, MN, USA; 5 BioTechnology Institute, College of Biological Sciences, University of Minnesota, Minneapolis, MN, USA; 6 Department of Pediatrics, the University of Oklahoma Health Sciences Center, Oklahoma City, OK, USA

## Abstract

The establishment of the gut microbiome in early life is critical for healthy infant development. Although human milk is recommended as the sole source of nutrition for the human infant, little is known about how variation in milk composition, and especially the milk microbiome, shapes the microbial communities in the infant gut. Here, we quantified the similarity between the maternal milk and the infant gut microbiome using 507 metagenomic samples collected from 195 mother-infant pairs at one, three, and six months postpartum. We found that the microbial taxonomic overlap between milk and the infant gut was driven by bifidobacteria, in particular by *B. longum*. Infant stool samples dominated by *B. longum* also showed higher temporal stability compared to samples dominated by other species. We identified two instances of strain sharing between maternal milk and the infant gut, one involving a commensal (*B. longum*) and one a pathobiont (*K. pneumoniae*). In addition, strain sharing between unrelated infants was higher among infants born at the same hospital compared to infants born in different hospitals, suggesting a potential role of the hospital environment in shaping the infant gut microbiome composition. The infant gut microbiome at one month compared to six months of age was enriched in metabolic pathways associated with *de-novo* molecule biosynthesis, suggesting that early colonisers might be more versatile and metabolically independent compared to later colonizers. Lastly, we found a significant overlap in antimicrobial resistance genes carriage between the mother’s milk and their infant’s gut microbiome. Taken together, our results suggest that the human milk microbiome has an important role in the assembly, composition, and stability of the infant gut microbiome.

## Introduction

The gut microbiome composition and maturation in early life plays an important role in the development of the immune system^1,2^, nutrient absorption^3,4^ and metabolism regulation^5^. Its assembly is influenced by microbes acquired from the mother^6–9^, among other sources^10^, starting from the first day of life^11^. While the impact of the maternal gastrointestinal, vaginal, oral and cutaneous microbial species on the infant gut microbiome assembly and development have been extensively investigated^6,11–15^, the role of the maternal breast milk microbiome in modulating infant gut microbes remains poorly understood.

Human breast milk represents, ideally, the sole source of nutrition for the infant in the first semester of life^16^. Nevertheless, milk remains heavily understudied, estimated to be less than 0.2% of all human-associated public metagenomic samples, based on data from 2021^17^. Maternal milk provides the infant with nutrients, such as proteins, lipids, carbohydrates (including human milk oligosaccharides), maternal immune cells, antibodies, and live bacteria^18–20^. The milk, and the bacteria that it carries, have therefore the potential to significantly impact infant gut microbiome composition, stability, and functionality. In addition, the presence of antimicrobial resistance genes in the milk microbiome can further influence the infant’s health and its gut microbiome composition^21^. The microbiome could also underlie the benefits of exclusive breastfeeding for a variety of chronic conditions including asthma^22,23^, childhood obesity^24^, type 1 diabetes^25^, and allergic disease^26^.

Despite its clear importance for infant health, research on the human milk microbiome has lagged behind studies of other human body sites. This is partly due to the nature of the sample itself: laced with human cells and high fat content, and characterized by low microbial biomass^27^, milk samples are challenging to process and sequence using standard approaches^27^. The few available milk microbiome cohorts are either limited by a small sample size or by lack of paired infant samples. Studies have also been hindered by the use of 16S rRNA gene sequencing^28,29^, which cannot reliably identify microbial strains^30^ and does not provide insights into the functional potential of the bacterial communities.

The primary objective of this study was to investigate the maternal milk microbiome in relation to the infant gut microbiome. This was achieved by collecting breast milk and infant stool samples in the first six months postpartum from a large cohort of healthy mothers and their infants, all of whom exclusively breastfed for at least one month. We used high-throughput shotgun metagenomics to assess species composition and stability over time, and to identify strain-sharing events between mother-infant pairs and between unrelated infants. We then analyzed the microbial functional potential, with particular focus on metabolic pathways and the antimicrobial resistance carriage of both maternal milk and infant stool samples over time.

## Results

### *Bifidobacterium longum* drives the compositional overlap between the infant gut and the maternal milk microbiomes

**Figure 1.**
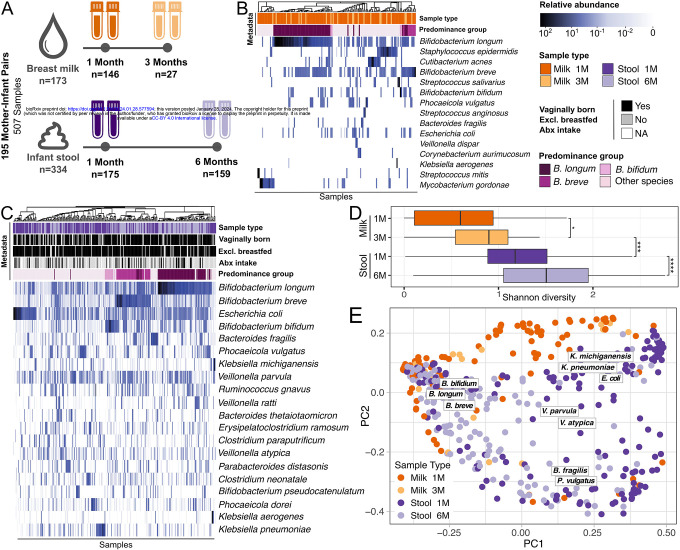
(A) Study design overview for the 507 samples collected from 195 mother-infant pairs. Infant stool samples (n=334) were collected at one and six months of life. Maternal breast milk samples (n=173) were collected one and three months after delivery. Taxonomic composition of (B) the most prevalent and abundant species found in the human breast milk and (C) in the infant gut microbiome samples in relation to sample collection time point, predominance group and other relevant infant metadata. Predominance group identifies the most abundant species in each sample. (D) Shannon diversity distribution for infant stool and maternal milk samples over time. P-values calculated using paired t-test (* p<0.05, ** p<0.01 and *** p<0.001). (E) Ordination plot based on Bray-Curtis distance between samples, colored by body site of origin and sampling time. Boxed species names indicate the species driving the clustering in that area of the PCoA and were obtained using the Weighted Averages Scores for species (see also Extended data 2).

We collected and sequenced 507 microbiome samples from 195 mother-infant pairs. All infants were born at term and were exclusively breastfed at one month of age. Breast milk was collected at one and three months postpartum (n=173), while infant stool samples were collected at one and six months postpartum (n=334, Fig. 1A). Infants were predominantly vaginally born (76%), exclusively breastfed at six months (66%), and antibiotics naive (66% had not received antibiotics by six months of age) (**Extended Data 1** and Suppl. Table 1). After shotgun sequencing, 34 samples were excluded from downstream analysis due to low sequencing yield. Species-level taxonomic profiling was performed with MetaPhlAn4^31^ (Suppl. Table 2).

The maternal milk microbiome was dominated by Bifidobacteria, in particular by *Bifidobacterium longum*, *Bifidobacterium breve* and *Bifidobacterium bifidum*, in order of prevalence (Fig. 1B and **Extended Data 2**). Other prominent members of the milk microbiome included skin-associated species such as *Staphylococcus epidermidis* and *Cutibacterium acnes*, oral-associated species such as *Streptococcus salivarius*, and gut-associated species such as *Escherichia coli and Phocaeiola vulgatus* (Fig. 1B). This is consistent with previous studies showing detecting skin-associated taxa in milk in addition to Bifidobacteria^32,33^.

The infant gut microbiome at one month was dominated by *E. coli*, *B. longum*, *B. breve*, *B. fragilis, B. bifidum*, *K. pneumoniae*, *Klebisella michiganensis*, and *Ruminoccus gnavus*, as well as species typically associated with the oral cavity, such as *V. parvula*, and *V. atypica* (Fig. 1C). *Phocaeiola vulgatus* and *E. coli* were among the species found in the infant gut as well as in the maternal milk (Fig. 1B–C). Bifidobacteria was the most prevalent and abundant genus in milk and stool samples at six months (**Extended Data 3A**). At the family taxonomic level, the prevalence of Enterobacteriaceae in the infant gut increased over time, while Bifidobacteriaceae showed the opposite trend (**Extended Data 3B**). Based on the dominant (most abundant) species, the infant stool samples could be divided in four groups, hereafter referred to as predominance groups: the first three dominated by *B. longum*, *B. breve*, and *B. bifidum*, respectively, and a fourth group dominated by species not belonging to the Bifidobacterium genus, mostly *E. coli, B. fragilis*, and *P. vulgatus* (Fig. 1C). 26.7% of infant stool samples were dominated by *B. longum*, 14.4% by *B. breve*, 4.5% by *B. bifidum* and 54.2% by non-bifidobacteria species (**Extended Data 4A**).

**Figure 2.**
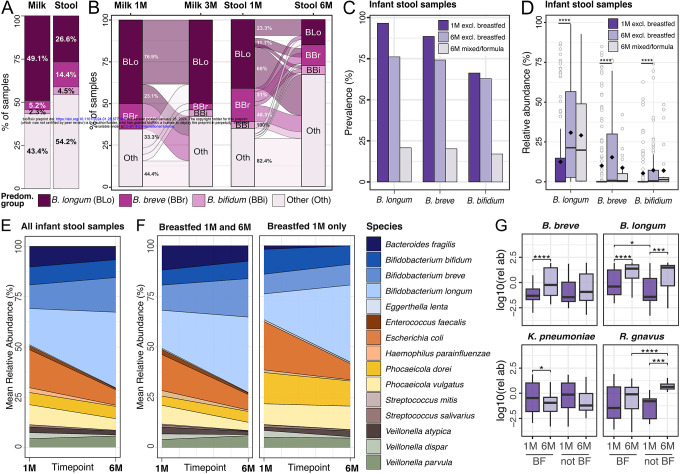
(A) Prevalence of each predominance group in milk and infant stool samples and (B) the transition of samples between predominance groups over time. Each sample is assigned one of four predominance groups indicated by the different colors. (C) Bifidobacteria mean prevalence and (D) distribution of relative abundances in exclusively breastfed infants at one and six months, and non-exclusively breastfed infants at six months of age. Black diamonds indicate the mean relative abundance per group. P-values calculated using Wilcoxon rank sum. Reported p-values are adjusted using Bonferroni method. (E) Species persistence in the infant gut across all samples and (F) stratified by breastfeeding at six months. (G) Relative abundance of some of the differentially abundant species between one and six months when divided by breastfeeding (BF) at six months. P-values calculated with paired t-test and adjusted with BH correction. **** for P ≤ 0.0001, *** for P ≤ 0.001, ** for P ≤ 0.01 and * for P ≤ 0.05.

Maternal milk was characterized by a significantly lower species richness than infant stool samples (p=7.4×10^−4^, paired t-test; Fig. 1D). Microbial diversity in infant stool samples increased significantly over time (p=2.4×10^−7^, paired t-test), while maternal milk showed a similar but milder trend (p=0.02, paired t-test; Fig. 1D). A principal component analysis showed that milk microbiome composition was partially overlapping with that of infant stool samples, especially those collected at six months of age (Fig. 1E). Such overlap was mostly driven by Bifidobacteria, in particular by *B. longum* (Fig. 1E and **Extended data 2**). Although all three Bifidobacterium strains were significantly correlated with the first principal coordinate, *B. longum* was more strongly correlated compared to *B. breve* and *B. bifidum* (Spearman’s correlation, R=−0.73, p<2.2×10^−16^; R=−0.4, p<2.2×10^−16^; and R=−0.2, p<5.3×10^−6^; respectively; Fig. 1E). Overall, in both milk and infant stool samples, *B. longum* was the most predominant and abundant species (Fig. 2A).

### *B. longum* in the infant gut becomes less prevalent over time and is associated with a more stable microbiome

To investigate the stability of the microbiome in our samples, we first focused on the above described predominance groups. In both breast milk and infant stool, *B. longum* was, among bifidobacteria, the species with the highest prevalence (49.1% and 26.6%, respectively) and highest relative abundance (15.1% and 12.5%, respectively) (Fig. 2A–B). Specifically, 49.1% of milk samples were dominated by *B. longum*, 5.2% by *B. breve*, and 2.3% by *B. bifidum* (Fig. 2A). The samples dominated by *B. longum* had the most stable microbiome over time: 76.9% of milk samples dominated by *B. longum* at one month maintained *B. longum* as the predominant species at three months, while the predominant species changed to non-bifidobacteria species in only 23% of samples. In contrast, the samples dominated by *B. breve* were characterized by the lowest stability, as all milk samples dominated by *B. breve* at one month switched to having a non-bifidobacteria as the dominant species at 3 months post-delivery (Fig. 2B). In infant stool samples at one month, 41.1% of samples were dominated by *B. longum*, while at three months that percentage decreased to 15.7% (Fig. 2B). Conversely, 35% of stool samples at one month were dominated by non-bifidobacteria, and this proportion increased to 67.1% at six months (Fig. 2B). 60% of samples dominated by *B. longum* at one month switched to being dominated by non-bifidobacteria species by six months, while 82.4% of samples dominated by non-bifidobacteria species at one month were still dominated by non- bifidobacteria at six months (Fig. 2B). Overall, the prevalence of bifidobacteria in the infant gut decreased over time, with a particularly pronounced reduction in infants that had ceased exclusive breastfeeding prior to six months of age (Fig. 2C). In contrast, when bifidobacteria were still present at six months, their relative abundance increased compared to the previous time point, irrespective of the breastfeeding practices at six months of age (Fig. 2D), and with considerable inter-personal variability (**Extended Data 4B**).

Next, we looked at the longitudinal stability of the microbiome composition at the species-level. We found that *B. longum* and *E. coli* were more abundant at six months compared to one month (BH-adjusted p=3×10^−8^ and p=1.5×10^−3^, respectively, t-test), while *V. dispar* decreased over time (BH-adjusted p=2.1×10^−3^, t-test; Fig. 2E). We then investigated if there were species that were differentially abundant between the two timepoints when stratifying the infants for exclusive breastfeeding. Infants that were exclusively breastfed through six months of age showed a significant increase in *B. longum*, *B. breve* and *E. coli* (BH-adjusted p=4×10^−8^, p= 1.9×10^−5^, and p=1.7×10^−3^, respectively, paired t-test; Fig 2F–G), and a significant reduction in *K. pneumoniae* and *V. dispar* (BH-adjusted p=0.04 and p=3.2×10^−6^, respectively, paired t-test; Fig 2F–G). Infants that were exclusively breastfed at one month but not at six months showed an increase in *R. gnavus* (BH-adjusted p=1.2×10^−3^, t-test) as well as *B. longum*, although at a lesser extent than infants exclusively breastfed through six months (BH-adjusted p=2.3×10^−4^, t-test; Fig 2F–G). Overall, the infant stool samples that were dominated by *B. longum* at both one and six months of age showed the most stable community composition over time (**Extended data 5**).

### Strain sharing is more common among unrelated infants born in the same hospitals

**Figure 3.**
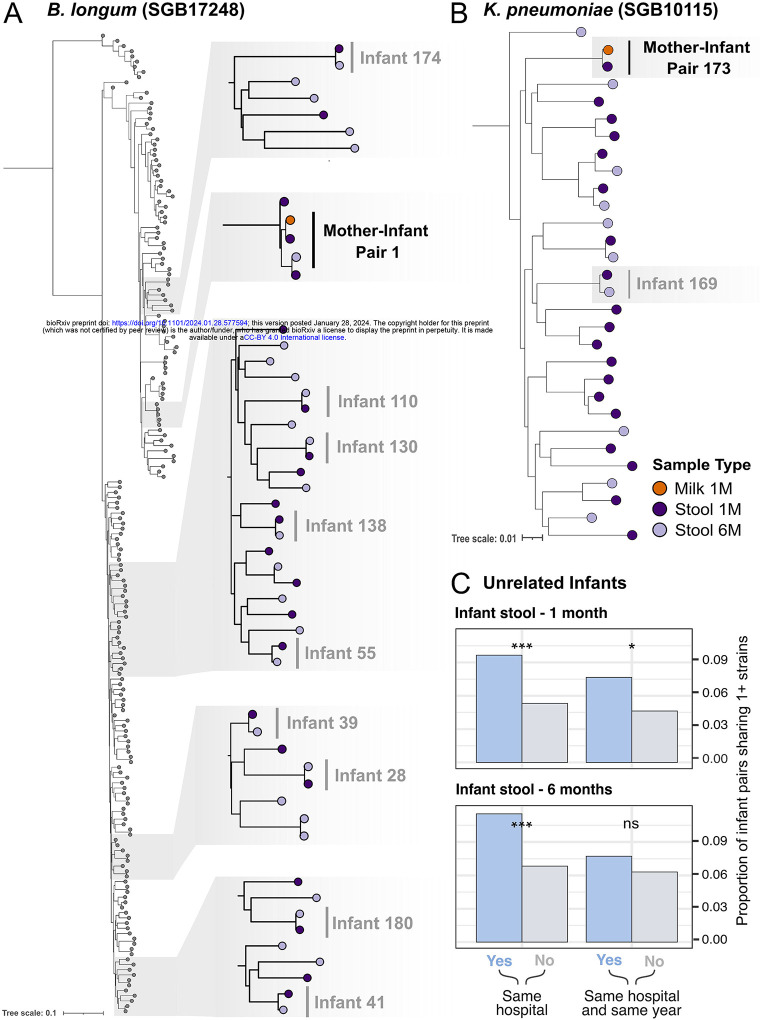
Strain sharing between maternal milk and infant stool samples for (A) the commensal species *B. longum* and (B) the pathobiont *K. pneumoniae*, highlighted in black. Instances of strain persistence within the same infant over time are highlighted with gray. (C) Proportion of unrelated infant pairs at one month (top) and six months (bottom) that share at least one strain (y-axis), considering infant pairs born in the same hospital (left), and same hospital as well as same year (right). Fisher’s exact test p-values are reported.

As species-level taxonomic profiling is not sufficient to identify potential transmission events, due to the genetic variability between conspecific strains^30^, we performed strain-level profiling with StrainPhlAn4^31^. This allows us to reliably identify strains that are found in pairs of samples, such as a mother and her infant or a pair of unrelated infants, defined as strain sharing. We reconstructed a total of 77 strains (Suppl. Table 3), of which 75 were found in the infant fecal samples and two in milk samples, due to the lower coverage. Out of the 43 strains present across multiple samples, we identified two instances of strain-sharing between a mother’s milk and her infant’s stools: the commensal species *B. longum* (in mother-infant pair 1), and the pathobiont *K. pneumoniae* (in mother-infant pair 173) (Fig. 3A–B). All strains identified in infant stool samples at one month were also identified at six months (Suppl. Table 4). Leveraging the multi-center nature of this cohort, we then compared strain sharing between unrelated infants born in the same hospital with that of infants born in different hospitals. We found that the proportion of unrelated infant pairs sharing at least one strain was significantly higher in infants born at the same hospital, compared to infants born in different hospitals at one month of age (p=9.6×10^−8^, Fisher’s Exact Test; Fig. 3C). This significant trend persisted also at six months of age (Fisher’s exact test p=9.7×10^−7^). When considering temporal overlap (same year of birth), in addition to spatial overlap (same hospital), we found that the proportion of infants born at the same hospital in the same year that shared at least one strain was higher than those born in the same year across different hospitals at one month (p=2.3×10^−3^, Fisher’s exact test) but not at six months (p=0.26, Fisher’s exact test; Fig. 3C).

### The maternal milk and early infant gut microbiomes are enriched in metabolic pathways for the biosynthesis of essential amino acids

**Figure 4.**
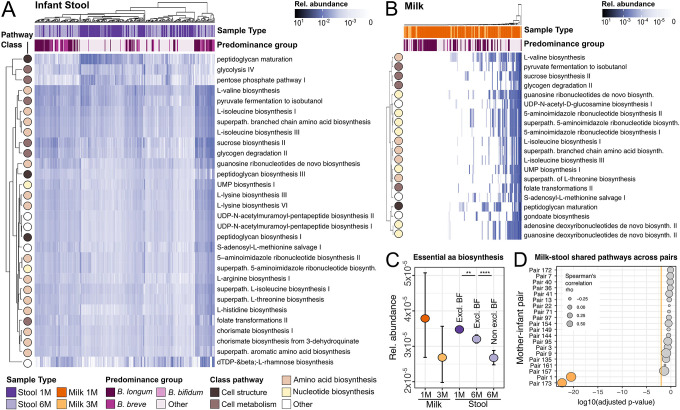
Most abundant pathways identified in (A) breastmilk at one and three months postpartum and (B) infant gut at one and six months of life. (C) Relative abundance of pathways involved in the biosynthesis of essential amino acids across sample type, collection time point and breastfeeding (BF). Error bars represent a 95% confidence interval calculated by bootstrapping (1000 times). P-values calculated using t-test, **** for P ≤ 0.0001 and ** p<0.01. Reported p-values are adjusted using Bonferroni method. (D) P-values of Spearman correlation between the abundance of all metabolic pathways shared between the maternal breast milk and infant stool samples for each mother-infant pair. P-values are corrected for multiple testing using Benjamin Hochberg correction and are shown in log10 scale. Only the top 20 mother-infant pairs are shown. Circle size denotes the correlation coefficient (rho), and the orange line denotes the significance threshold (log10 of p-value=0.01).

Next, we investigated the functional potential of the maternal milk and infant gut microbiomes using HUMAnN3^34^, a method that profiles microbial metabolic pathways from metagenomic data. The most prevalent pathways identified in the infant stool samples were associated with de-novo biosynthesis of molecules, in particular of essential amino acids (such as valine, isoleucine, threonine, and lysine) and ribonucleotides (Fig. 4A). Other abundant pathways were associated with cell structure (i.e. peptidoglycan maturation), and energy metabolism. The pathways involved in peptidoglycan maturation were present at higher relative abundances in infant stool samples at one month compared to six months (p=1.3×10^−14^, t-test; Fig. 4A), and this increase was particularly prominent for samples dominated by non-bifidobacteria species (**Extended Data 6A**). In milk, the most prevalent and abundant pathways were associated with the biosynthesis of nucleotides and amino acids (such as valine and isoleucine), cell metabolism (sucrose biosynthesis, glycogen degradation, pyruvate fermentation and folate transformations), and cell structure (peptidoglycan maturation) (Fig. 4B). In infant stool samples, the pathways associated with essential amino acid biosynthesis were significantly more abundant at one month compared to six months, and this trend was significantly more pronounced for infants that were not exclusively breastfed anymore at six months (Bonferroni-adjusted p=5.2×10^−3^ for infants exclusively breastfed at one and six months, and p=4.9×10^−5^, for infants exclusively breastfed at one month only, t-test; Fig. 4C). In particular, milk samples at one month dominated by *B. longum*, but not other Bifidobacteria, were associated with the highest abundance of metabolic pathways associated with the biosynthesis of essential amino acids (**Extended Data 6B**).

We then sought to investigate the relationship between the abundance of the metabolic pathways identified from microbes within each mother’s milk with those identified in her infant’s stool samples. We found a significant correlation between the metabolic pathways found in breast milk with those found in infant stool samples only for the mother-infant pairs for which we previously identified strain sharing events (BH-adjusted p=4.3×10^−23^ and p=3.4×10^−21^, for mother-infant pair 1 and 173, respectively, Spearman’s correlation; Fig. 4D and **Extended data 7**). When investigating specific pathways, rather than specific mother-infant pairs, we find that no pathway showed a significant correlation in its abundance in milk compared to infant stool samples (**Extended Data 8**).

### The infant gut and the maternal milk microbiomes harbor a diverse landscape of antimicrobial resistance genes

**Figure 5.**
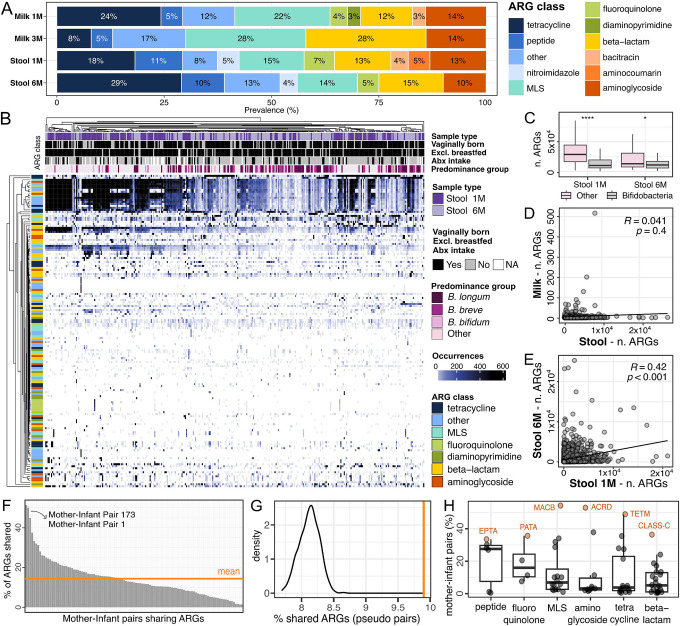
(A) Prevalence of the antimicrobial resistance genes (ARGs) classes predicted in maternal milk and the infant gut microbiome over time, calculated as percentage over the total number of detected ARGs for each sample type. (B) ARGs carriage in infant stool samples, divided by collection time point, infant metadata, predominance group and ARGs class. (C) Number of detected ARGs in infant stool samples dominated by bifidobacteria and non bifidobacteria species at one and six months of life. P-values calculated using t-test, **** for P ≤ 0.0001 and * for P ≤ 0.05. (D) Correlation between ARG carriage in maternal milk and infant stool samples, and (E) between infant stool samples at one month and six months. Each dot is a combination of a mother-infant pair and a predicted ARG class. Spearman’s R-values (correlation coefficient) and p-values are reported. (F) Percentage of ARG genes shared between at least one maternal and one infant sample, for each mother-infant pair. Mean value is indicated by the orange line. The mother-infant pairs for which strain sharing events were identified are highlighted with the arrow. (G) Distribution of the percentage of shared ARGs between at least one maternal milk and one infant stool sample on permuted mother-infant pairs (pseudo-pairs). The mean value obtained from real mother-infant pairs is indicated by the orange line. (H) Percentage of mother-infant pairs sharing antimicrobial resistance genes, divided by ARG class. For each ARG class, percentage value was calculated on the total number of mother-infant pairs in which that ARG class was identified in at least one sample. The most frequently shared antimicrobial resistance genes per class are highlighted in orange.

The gut is a reservoir of antimicrobial resistance, yet its composition and longitudinal variability remain poorly characterized in infants^35,36^. Even less is known about the antimicrobial resistance genes (ARGs), collectively known as the resistome^37^, present in human breast milk, and their transmission to the infant during lactation^21,38–40^. To investigate the resistome in the maternal milk and the infant gut, we used DeepARG^41^, which leverages deep learning to predict antimicrobial resistance genes from metagenomic data. We then compared ARG classes across sample types and collection timepoints. Maternal milk and infant stool differed in terms of ARGs classes composition and prevalence (Fig. 5A, **Extended Data 9A**). In the milk, the most prevalent (25% on average) antimicrobial resistance class identified was against macrolide-lincosamide-streptogramin (MLS). Overall, maternal milk was characterized by a lower diversity of detected ARG classes compared to infant stool samples (p=1.4×10^−14^, t-test; **Extended Data 9B-C**). In both milk and infant stool samples, the diversity of ARGs classes increased over time, although this increase was statistically significant only in the milk (p=2.4×10^−3^ and p=0.61 for milk and stool samples respectively, paired t-test; **Extended Data 9C**).

The infant gut resistome was mostly dominated by resistance to tetracycline, MLS, aminoglycoside, and beta-lactams (Fig. 5A). Infants whose stool samples were dominated by bifidobacteria were characterized by a significantly lower carriage rate of ARGs (p=7.6×10^−12^ and 4.2×10^−2^ at one and six months respectively, t-test; Fig. 5B–C). We found no significant difference in the resistome of infants that were born via C-section compared to those born via vaginal delivery (p=0.34 and p=1 at 1 month and 6 months, respectively, t-test), between those exposed to antibiotics and those that were antibiotics-naive (p=0.3 and p=0.18 at one month and six months, respectively, t-test), nor between those exclusively breastfed at six months of age and those fed with a mixture of breast milk and formula (p=0.13, t-test; **Extended Data 9D-F**). Nevertheless, we found extensive ARGs carriage in infants with no recorded pre-, during-, and postpartum exposure to antibiotics (Fig. 5B).

When comparing the overall resistome across all mothers and infants, we found no significant correlation between the ARGs found in milk and the ARGs found in the infant stool samples (R=−0.041, p=0.4, Spearman’s test; Fig. 5D). However, the infant gut resistome at one month was positively correlated with the resistome at six months of age (R=0.42, p=8.7×10^−48^, Spearman’s test; Fig. 5E).

Considering sharing of ARGs between milk and stool samples within each mother-infant pair, we found that mother-infant pairs shared a significantly higher number of ARGs than what was expected by chance (p<0.001, by permutation analysis, see Methods; Fig. 5F–G). Mother-infant pairs 1 and 173, for which strain sharing events were identified, were the pairs with the highest rate of shared ARGs between the mother’s milk and her infant stool samples (Fig. 5F). On average, the most commonly shared antimicrobial resistance classes were associated with resistance to peptides, fluoroquinolone, and MLS, while the most commonly shared antimicrobial resistance genes were MACB (MLS class), ACRD (aminoglycoside class), and TETM (tetracycline class) (Fig. 5H).

## Discussion

Although breast milk represents a critical source of nutrition for the developing infant, little is known about the milk microbiome, how it changes over time, its metabolic potential, and how it shapes the infant’s gut microbiome – all questions necessitating the use of high resolution sequencing techniques, such as metagenomics. Here, we investigated the composition, strain sharing, functional potential, and antimicrobial resistance of the maternal breast milk and the infant gut microbiome in early life in predominantly exclusively breastfeeding mother-infant dyads. In line with previous studies, the milk microbiome yielded a lower number of reads and was characterized by a reduced microbial diversity compared to the infant gut microbiome^11,21,27^. The milk at one month postpartum, considered largely mature^18^, was dominated by bifidobacteria, in particular *B. longum*, *B. breve*, and *B. bifidum*. The presence in the milk of typical oral species is likely due to the possible transfer of microbes from the oral cavity of the infant to the breast milk during suckling, a process called retrograde flow^42^. Bifidobacteria also dominated the gut microbiome of the infants, driving overlap in taxonomic composition between the maternal milk and infant gut microbiomes. In particular, infant stool samples largely clustered by which Bifidobacterium species was most abundant. We broadly identified 4 predominance groups, dominated by *B. longum*, *B. breve* and *B. bifidum* respectively, and a fourth group which included the samples dominated by other non bifidobacteria species, most commonly *E. coli*. While *B. longum*, *B. bifidum*, and *B. breve* were often coexisting and present at comparable abundances in milk, these species were largely mutually exclusive in the infant gut. These results suggest a higher level of competition between bifidobacteria species in the infant gut compared to the maternal milk. Bifidobacteria, most commonly found in the human gut^43^ and maternal milk^44^, are known key foundation taxa of the infant gut microbiome and their abundance is primarily shaped by breast milk intake^45–47^. Indeed, the stools from exclusively breastfed infants had higher prevalence and abundance of bifidobacteria compared to those that ceased exclusive breastfeeding prior to six months of age. Considering change over time, bifidobacteria increased in relative abundance from one to six months of age, in line with what was shown by Heisel et al.^48^. This pattern was more pronounced in infants exclusively breastfed through six months.

We found two cases of strain sharing between the mother’s milk and her infant’s gut microbiome, specifically for the commensal species *B. longum*, and for the pathobiont *K. pnuemoniae*. Despite *K. pneumoniae* potential association with silent sepsis in infants^49,50^ and with mastitis and milk loss in cows^51^, the infants in our cohort showed no clinical manifestations nor had any of the mothers reported mastitis/breast inflammation at the time of their study visit and milk collection. While strain sharing between the maternal and the infant gut has been extensively investigated^8,11^, only one metagenomic study has so far, to the best of our knowledge, successfully identified strains shared between the maternal breast milk and the infant gut microbiome^52^. Our results provide evidence that strain sharing between the infant gut and the maternal breast milk occurs, even if at a rate considerably lower than what was previously found between the infant and the maternal gut^11^. The fact that we were able to reconstruct only two strains in our milk samples, and yet both were found to be shared between mother’s milk and infant gut, indicates that our estimate for strain sharing between breast milk and infant gut is likely an underestimate due to the low sequencing yield of the milk samples. This is a common challenge with this type of sample^27^.

We found an enrichment of strain sharing events among unrelated infants born in the same hospital compared to those born in different hospitals, a trend that still persisted at six months of age. Strain sharing between unrelated infants was previously reported in hospitalized premature infants, but this represents, as far as we know, the first evidence of microbial strain sharing between healthy, at-term infants, months after hospital discharge^53,54^. This result suggests that the short-term postpartum hospital stay might play a role in the infant’s gut strain acquisition and persistence over time. An alternative hypothesis could be that infants born in the same hospital live in the same geographic area and can acquire microbiomes through other shared environments, such as daycare facilities. However, we could not test this hypothesis as these high-resolution geographical and environmental metadata were not collected.

Resistome analysis showed that both the infant gut and the maternal milk harbor a diverse landscape of antimicrobial resistance gene classes, mostly dominated by resistance genes for tetracycline, aminoglycoside, and macrolide-lincosamide-streptogramin, even in infants that had no recorded exposure to pre-, intra-, and post-partum antibiotics. Tetracycline is not prescribed in pregnancy and its use is not recommended in children younger than 8 years due to potential permanent discoloration of teeth^55^. Still, tetracycline resistance was the most abundant antibiotic resistance class in both milk and infant stool samples at one month. Aminoglycoside (e.g. gentamicin and streptomycin) and macrolides (e.g. azithromycin and erythromycin) are widely used in the perinatal period^40^, therefore resistance is potentially acquired via antibiotic exposure. Milk and infant stool samples at one month were characterized by higher prevalence and diversity in resistance classes, compared to later time points. In addition, the majority (70%) of ARGs found in the infant gut at one month were associated with predominance of non-bifidobacteria species, in particular *E. coli*. The presence of antibiotic resistance in the newborn gut has been reported in previous studies^35,36,56^, with indications of resistance transmission from the maternal gut to the infant gut via mobile genetic elements^57^. Our results show a significant overlap between the resistome of infants and that of their mother’s milk, and that this overlap was particularly pronounced when evidence of strain sharing was found. This suggests that the infant gut resistome is likely influenced to some degree by acquisition from breastmilk, in addition to other mechanisms, such as mobile genetic elements, vertical strain transmission and environmental exposure, and from other maternal body sites, such as gut^57^.

This study has several limitations. Milk sampling was performed via the use of breast pumps, which could potentially impact milk microbiome composition^58^. In addition, the low number of three-month milk samples as compared to one-month milk samples limits our conclusions on milk microbiome composition at three months and strain-sharing with the infant beyond one month postpartum. The low sequencing yield for breast milk samples limited our power for detection of strain sharing between milk and infant gut, suggesting that the sharing patterns are an underestimate. In addition, as we sampled only milk and infant stool samples, we could not confirm that the strain sharing events we identified were indeed cases of microbial transmission from the maternal milk to the infant gut, rather than cases of strain acquisition by both the mother and the infant from external sources not investigated in this study. Finally, infants were born across multiple hospitals, which usually represents a limitation, as it could potentially influence the microbial composition. However, all samples were collected following the same procedures and the samples’ taxonomic composition did not cluster based on the sampling location. Furthermore, the multi-center structure of this study enabled us to quantify strain sharing among unrelated individuals across different hospitals.

In this work, we characterized the microbiome composition, function and antimicrobial resistance potential of the breast milk of mothers and the gut microbiome of their infants during the first six months postpartum. We found evidence of strain- and antimicrobial resistance gene-sharing between mother-infant pairs. Taken together our results indicate that the maternal breast milk plays a role in infant gut microbiome and resistome establishment, development and temporal stability. Our results represent an important step towards the strain-level characterization of the maternal milk microbiome in relation to the infant’s gut and its better representation in public repositories.

## Methods

### Sample collection

The participants from this study were enrolled as part of the Mothers and Infants Linked for health (MILk) cohort^59–61^. Recruitment, clinical metadata and sample collection were performed as previously described in^59–61^. All mothers were enrolled prenatally from the University of Minnesota in collaboration with HealthPartners Institute (Minneapolis, MN) and were provided written informed consent. All relevant guidelines and regulations were observed. Inclusion criteria included a healthy, uncomplicated pregnancy and the intention to exclusively breastfeed the infant. All mothers were aged between 21–45 years, were not diabetic and non-smokers, and delivered a full-term infant. No case of breast infection or mastitis was reported during the milk sample collection. All infants were singletons and born at term with a birth weight that was appropriate for their gestational age, and were exclusively breastfed to at least one month of age (Supplementary Table 1 and **Extended Data 1**). All relevant metadata were collected via the hospital’s electronic medical health records and via questionnaires during sample collection (Supplementary Table 1 and **Extended Data 1**). Breast milk samples were collected at one and three months postpartum. Milk collection was performed as follows: mothers fed their infant from one or both of their breasts, until the infant was satisfied. After two hours, the milk was collected from the right breast using a hospital grade electric breast pump (Medela Symphony; Medela, Inc., Zug, Switzerland), until cessation of production. Each milk sample was gently mixed, and its volume and weight were recorded. Aliquots were stored at −80 °C within 20 minutes of collection and kept at that temperature until RNA/DNA extraction. Infant stool samples were collected at one and six months of age. Stool samples collection, storage, and associated metagenomic shotgun DNA extraction were performed as described in previous works^59–62^. Stool samples were either collected from diapers during a study visit or at home by the mother. In case of collection during a study visit, the sample was immediately frozen at −80°C, while in case of home collection the sample was stored in 2 ml cryovials with 600 μl RNALater (Ambion/Invitrogen, Carlsbad, CA), and later stored at −80°C upon arrival to the lab at the University of Minnesota.

### DNA extraction and metagenomic sequencing

DNA extraction was performed with PowerSoil kit (QIAGEN, Germantown, MD), eluted with 100 μl of the provided elution solution, and stored in microfuge tubes at −80°C. The extracted DNA was used to construct libraries for metagenomic shotgun sequencing using the Illumina Nextera XT 1⁄4 kit (Illumina, San Diego, CA, United States). Metagenomic shotgun sequencing libraries were sequenced on an Illumina NovaSeq system (Illumina, San Diego, CA) using the S4 flow cell with the 2×150 bp paired end V4 chemistry kit by the University of Minnesota Genomics Center.

### Quality filtering and removal of human reads

Host DNA was removed using paired-end mapping with Bowtie2^63^ version 2.2.4 against a human reference genome hg38. Unmapped paired-end reads were filtered using SAMtools^64^ version 1.9 with following parameters “samtools view -bS, samtools view -b -f 12 -F 256, samtools sort -n -m 5G -@ 2, samtools fastq -@ 8 −0 /dev/null -s /dev/null -n”. BEDtools^65^ was then used to convert the bam files to fastq files containing the non-human paired-end reads. Then, adapter sequences were removed and the samples were filtered and trimmed using the default parameters of Trimmomatic^66^. Host DNA content in breast milk samples was 74.13 ± 16.37 %. FastQC^67^ was used to analyze the quality of the metagenomic reads. Read lengths were 1,224,953 and 1,224,953 bp for the forward and reverse reads, respectively. Mean PHRED of the score was 33. Samples with less that had 500 or less reads that mapped to the Metaphlan4^31^ database (mpa_vJan21_CHOCOPhlAnSGB_202103) were discarded. A total of 34 of the 507 samples were excluded from downstream analysis. After pre-processing, milk samples yielded 2.45±1.36 million reads per sample, while stool samples yielded 10.8±3.96 million reads per sample (**Extended Data 10**).

### Species- and strain- level taxonomic profiling and strain sharing

Species-level taxonomic profiling was based on marker genes using MetaPhlAn4^31^, with the following parameters: “--bt2_ps sensitive” text. MetaPhlAn4 infers taxonomic prevalence and abundance by using unique marker genes for 26,970 species-level genome bins^31^. Merged abundance table was created using the MetaPhlAn4 utils script. Contaminants found in the blank samples as well as additional known contaminant species were removed before downstream analysis. MetaPhlAn4 profiles are available in Supplementary Table 2. Heatmaps for species-level taxonomic composition were generated with ComplexHeatmaps^68,69^, using euclidean distance for hierarchical clustering. Strain-level profiling was performed using StrainPhlAn4^31^, based on single-nucleotide variant calling, with the following parameters: “--mutation_rates --trim_sequences 50 --marker_in_n_samples 50 -- secondary_sample_with_n_markers 50 --breadth_thres 50”. Strain_transmission.py output (Supplementary Table 3) was used to identify strain sharing events among related and unrelated individuals. Phylogenetic trees were visualized with iTOL^70^.

### Alpha and beta diversity

Alpha diversity was computed using the MetaPhlAn4 utility script calculate_diversity.R. The ordination plot was computed from the MetaPhlAn4 relative abundances using the “vegan” R package (v2.6–4).

### Functional profiling

Functional prediction was performed with HUMAnN3^34^. Biosynthetic potential of essential amino acids were calculated by searching the output pathways for the following keywords: “L-histidine biosynthesis”, “L-isoleucine biosynthesis”, “L-isoleucine biosynthesis”, “L-lysine biosynthesis”, “L-methionine biosynthesis”, “L-phenylalanine biosynthesis”, “L-threonine biosynthesis”, and “L-valine biosynthesis”. Profiles relative abundance plots show 95% confidence interval using bootstrapping with 1000 repetitions using the Hmisc R library (v5.0–1). Raw functional profiles are available in Supplementary Table 5.

### Antimicrobial resistance genes prediction

Antimicrobial resistance genes were predicted from raw metagenomic short reads using DeepARG v1.0.2^41^. DeepARG leverages deep learning models to identify over 30 antimicrobial resistance classes. Predicted resistance is classified as “predicted” or “potential” by the models. To reduce false positives detection, only “predicted” resistance, with a minimum identity threshold of 95% of the target sequence was included in the downstream analysis. ARG predictions classified as “multidrug” or “unclassified” were excluded from downstream analyses. Raw DeepARG profiles are available in **Supplementary Table 6**. Permutation analysis in Figure 5G was performed as follows: maternal milk sample names were randomly permuted, while data and the infant sample names were preserved, generating pseudo-mother-infant pairs, and the mean number of antimicrobial resistance genes shared between the pseudo-mother-infant pairs was calculated. This was repeated 1000 times, and the distribution of the mean values obtained from pseudo-mother-infant pairs was compared to the mean value obtained from the real mother-infant pairs.

### Statistical analysis

Statistical analysis was done in R^71^ version 4.2.2 (2022–10-31). All figures if not indicated otherwise were drawn with ggplot^72^ version 3.4.1. All analyses have been performed with open source software referenced in the Methods section. In Fig. 3C, Fisher’s exact test was calculated on the occurrences tables defined as follows: number of unrelated infant pairs sharing at least one strain (Yes/No) versus infant pairs born in the same hospital (Yes/No). Fisher’s exact test and occurrence tables were calculated in a similar fashion when considering the same hospital and the same window of birth (same month), and separately for infant pairs at one and six months of age.

## Supplementary Material

Supplement 1

Supplement 2

Supplement 3

Supplement 4

Supplement 5

Supplement 6

Supplement 7

Supplement 8

Supplement 9

Supplement 10

Supplement 11

Supplement 12

Supplement 13

Supplement 14

Supplement 15

1

## Data Availability

The raw metagenomic sequences and the associated metadata were deposited and are available on NCBI Sequence Read Archive (SRA) under the BioProject accession number PRJNA1019702. Comprehensive metadata are available in the Supplementary Material. Code is available on git https://github.com/blekhmanlab/milk_infant_microbiome.
