# Supplementary figures and images for: Assembly, stability, and dynamics of the infant gut microbiome are linked to bacterial strains and functions in mother’s milk
Source: bioRxiv. 2024 Jan 28:2024.01.28.577594. Preprint. [Version 1] doi: 10.1101/2024.01.28.577594 (PMC10849666; doi:10.1101/2024.01.28.577594)

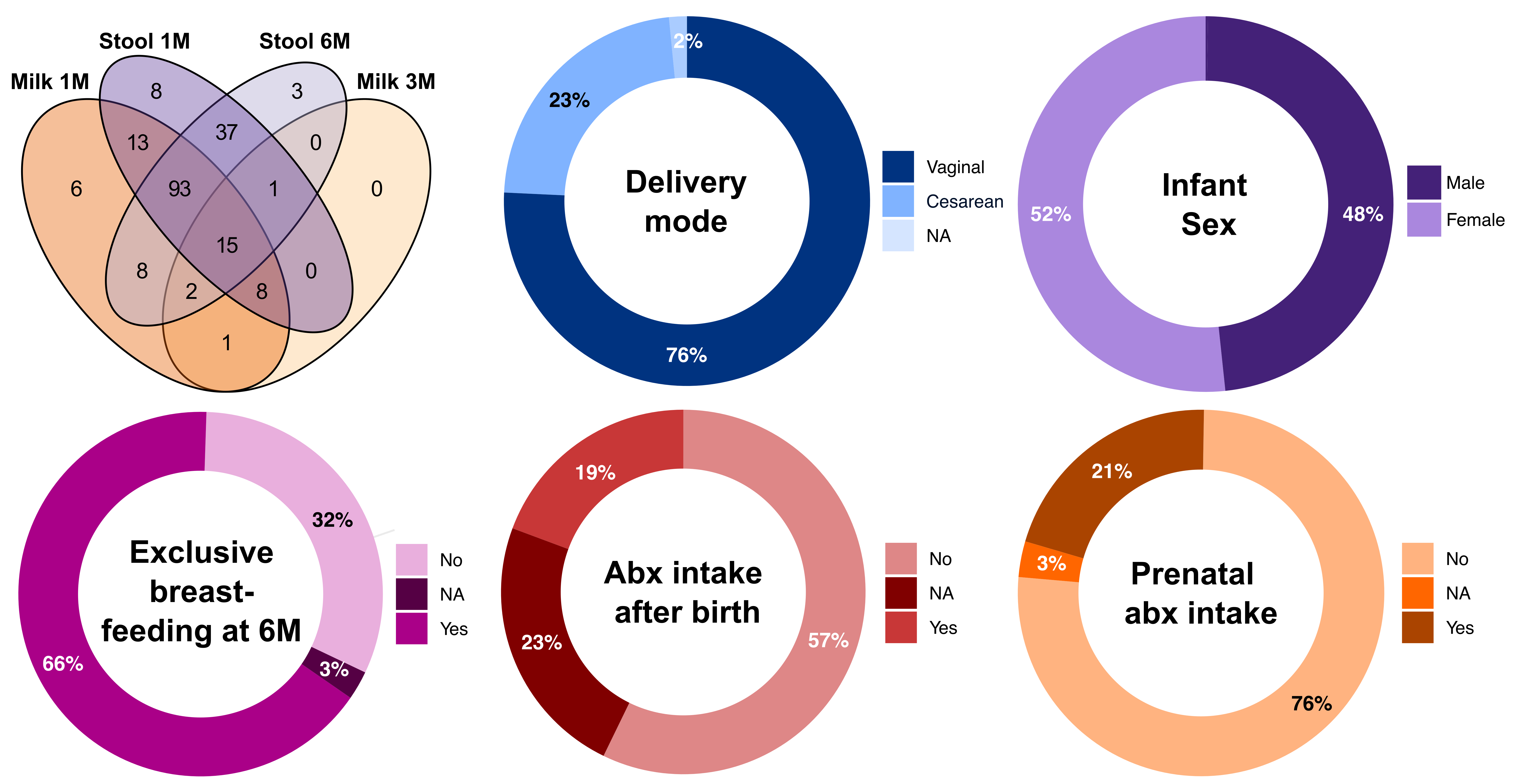

Supplement: Supplement 1 [file media-1.tif]

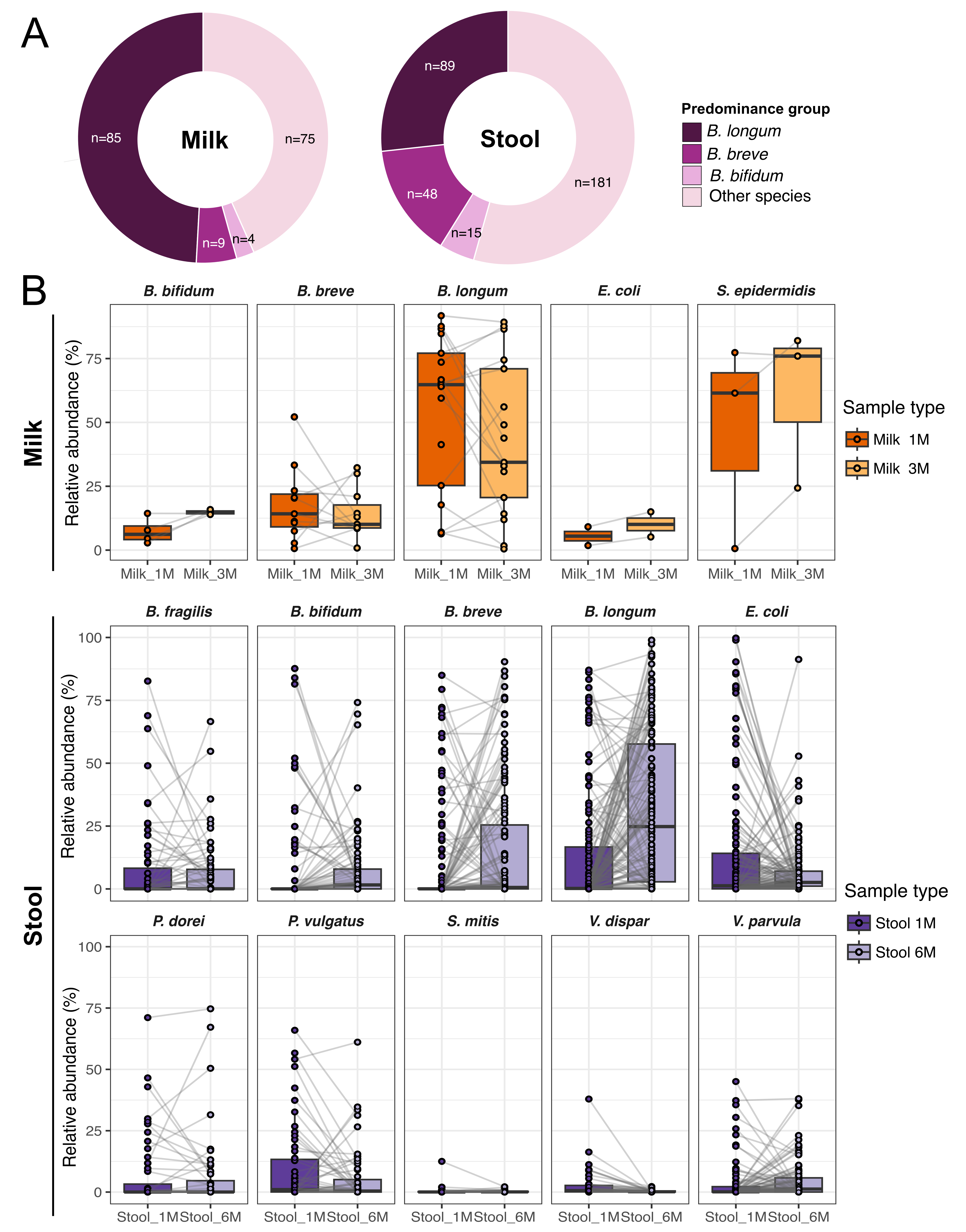

Supplement: Supplement 4 [file media-4.tif]

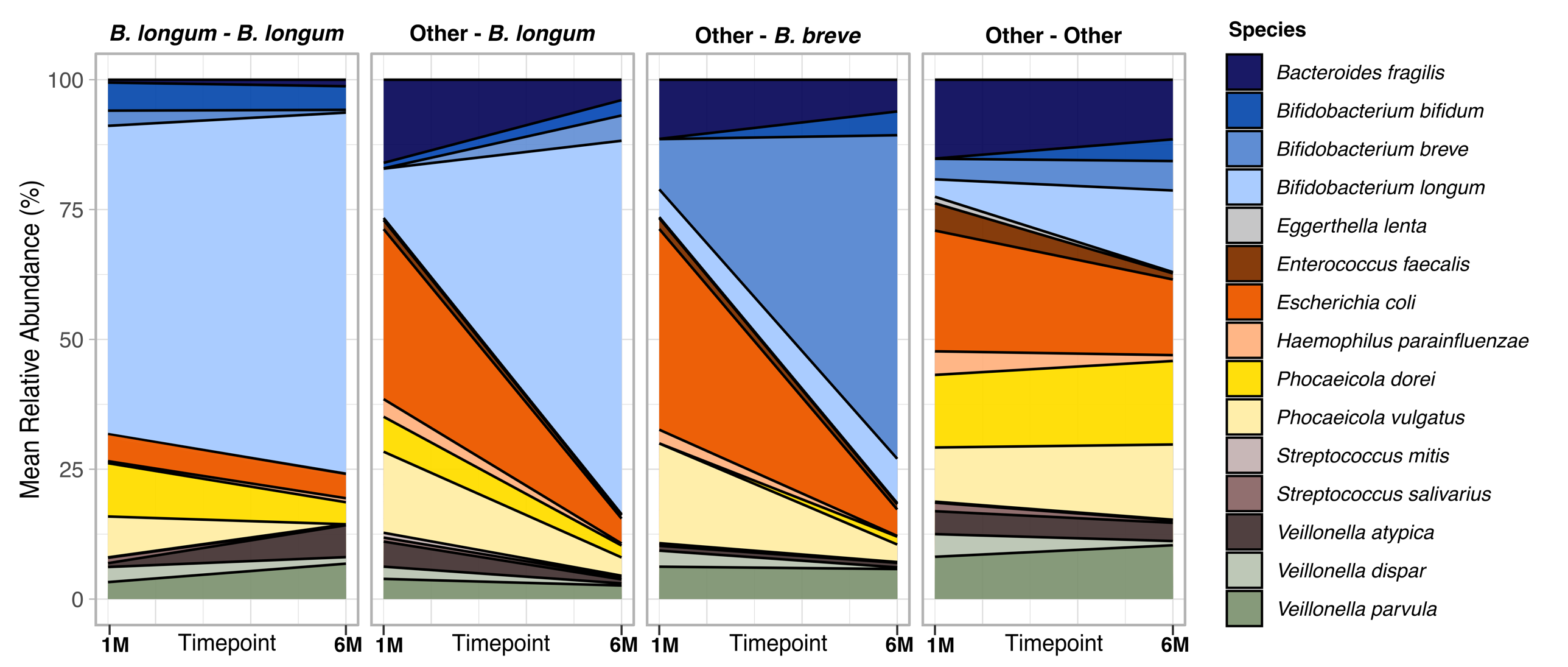

Supplement: Supplement 5 [file media-5.tif]

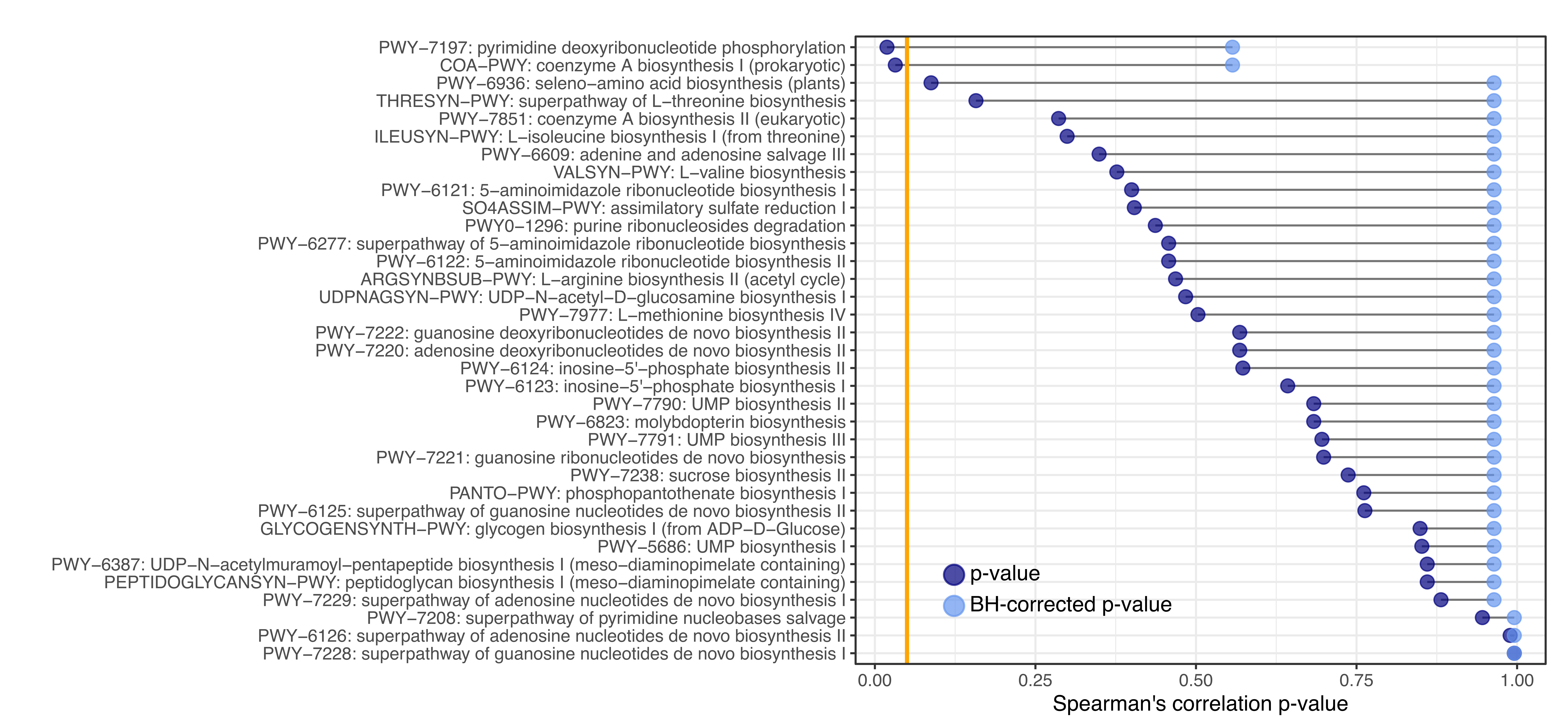

Supplement: Supplement 7 [file media-7.tif]

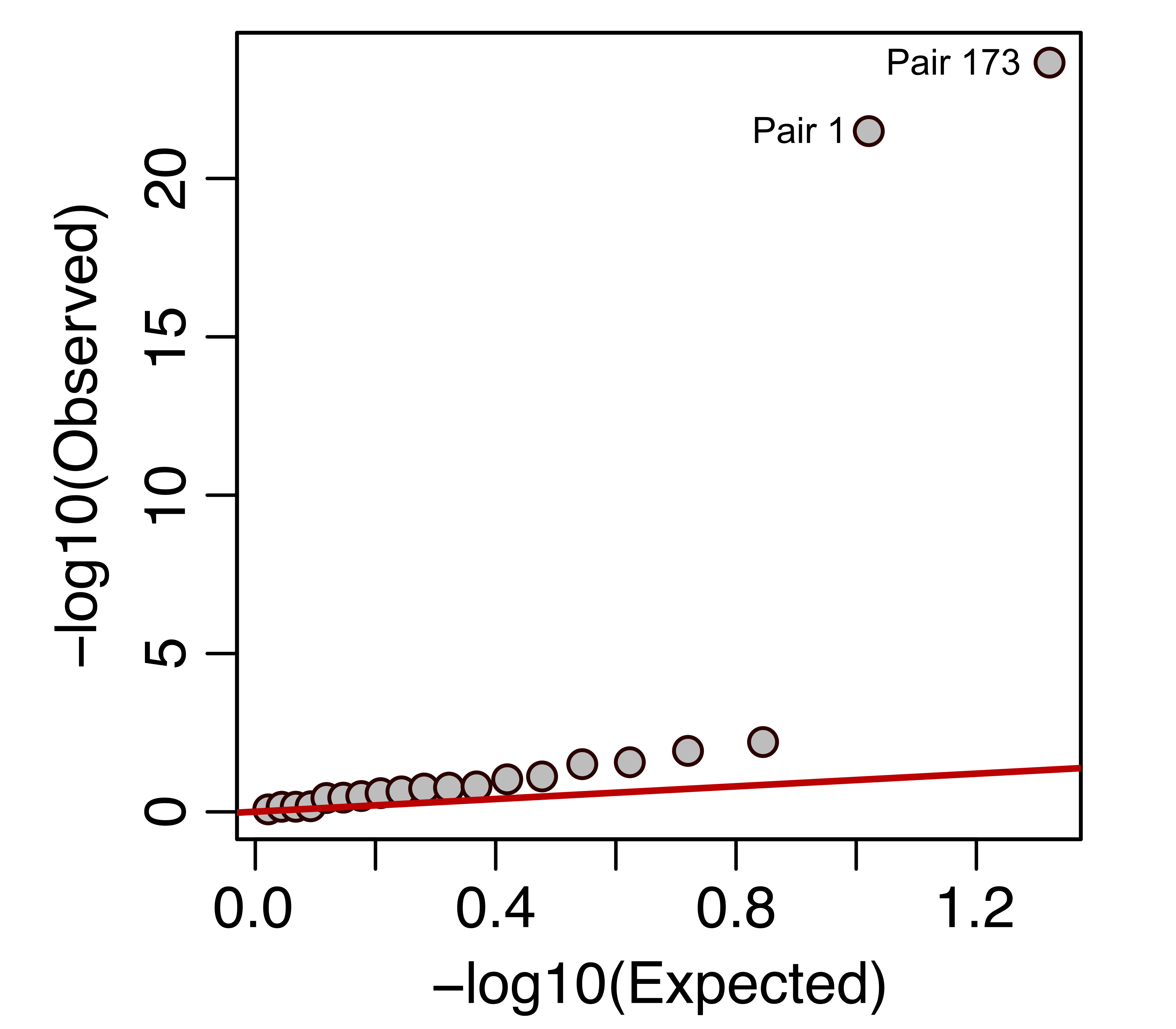

Supplement: Supplement 8 [file media-8.tif]
