# Supplementary material for: Assembly, stability, and dynamics of the infant gut microbiome are linked to bacterial strains and functions in mother’s milk
Source: bioRxiv. 2024 Jan 28:2024.01.28.577594. Preprint. [Version 1] doi: 10.1101/2024.01.28.577594 (PMC10849666; doi:10.1101/2024.01.28.577594)
Supplement: 1 [file NIHPP2024.01.28.577594V1-supplement-1.pdf]

bioRxiv preprint doi: https://doi.org/10.1101/2024.01.28.577594; this version posted January 28, 2024. The copyright holder for this preprint (which was not certified by peer review) is the author/funder, who has granted bioRxiv a license to display the preprint in perpetuity. It is made available under aCC-BY 4.0 International license.

Supplementary Material

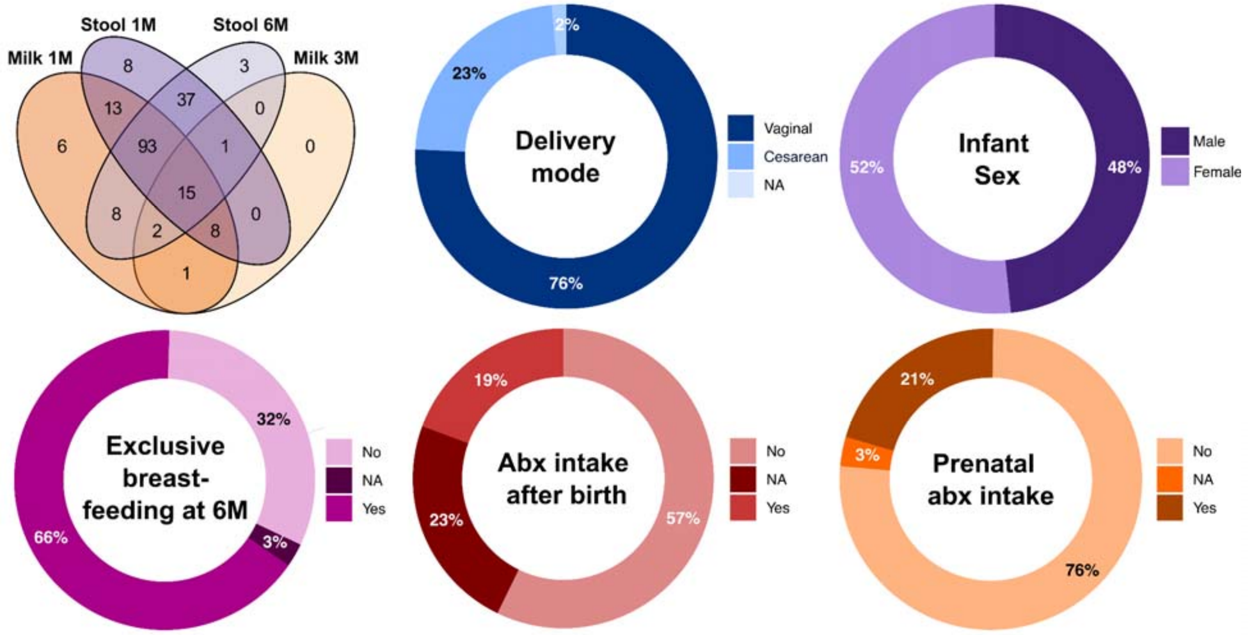

Extended Data 1. Number of mother infant pairs for which samples were collected across body sites and collection timepoints, singularly taken or in combinations. Relevant metadata available for the MILk cohort, including delivery mode, infant sex, exclusive breastfeeding at 6 months of age, and antibiotic (abx) intake pre- and after-partum. All infants were exclusively breastfed at 1 month of age.

bioRxiv preprint doi: https://doi.org/10.1101/2024.01.28.577594; this version posted January 28, 2024. The copyright holder for this preprint (which was not certified by peer review) is the author/funder, who has granted bioRxiv a license to display the preprint in perpetuity. It is made available under aCC-BY 4.0 International license.

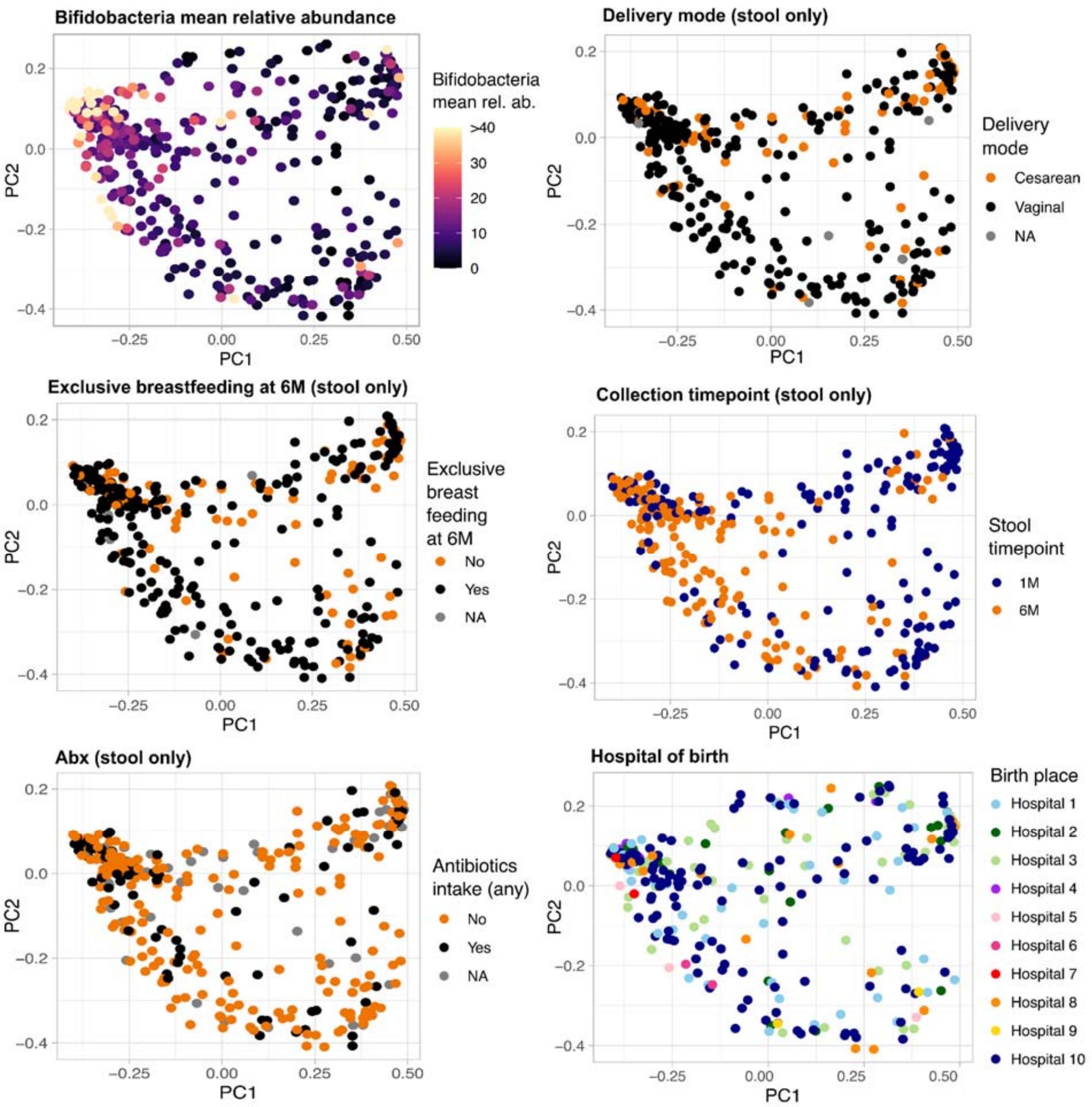

Extended Data 2. Ordination plot coloured by relevant metadata. All body sites and collection time points are included unless otherwise specified. PCoA of hospitals includes only the samples for which the birth hospital or clinic name is known.

bioRxiv preprint doi: https://doi.org/10.1101/2024.01.28.577594; this version posted January 28, 2024. The copyright holder for this preprint (which was not certified by peer review) is the author/funder, who has granted bioRxiv a license to display the preprint in perpetuity. It is made available under aCC-BY 4.0 International license.

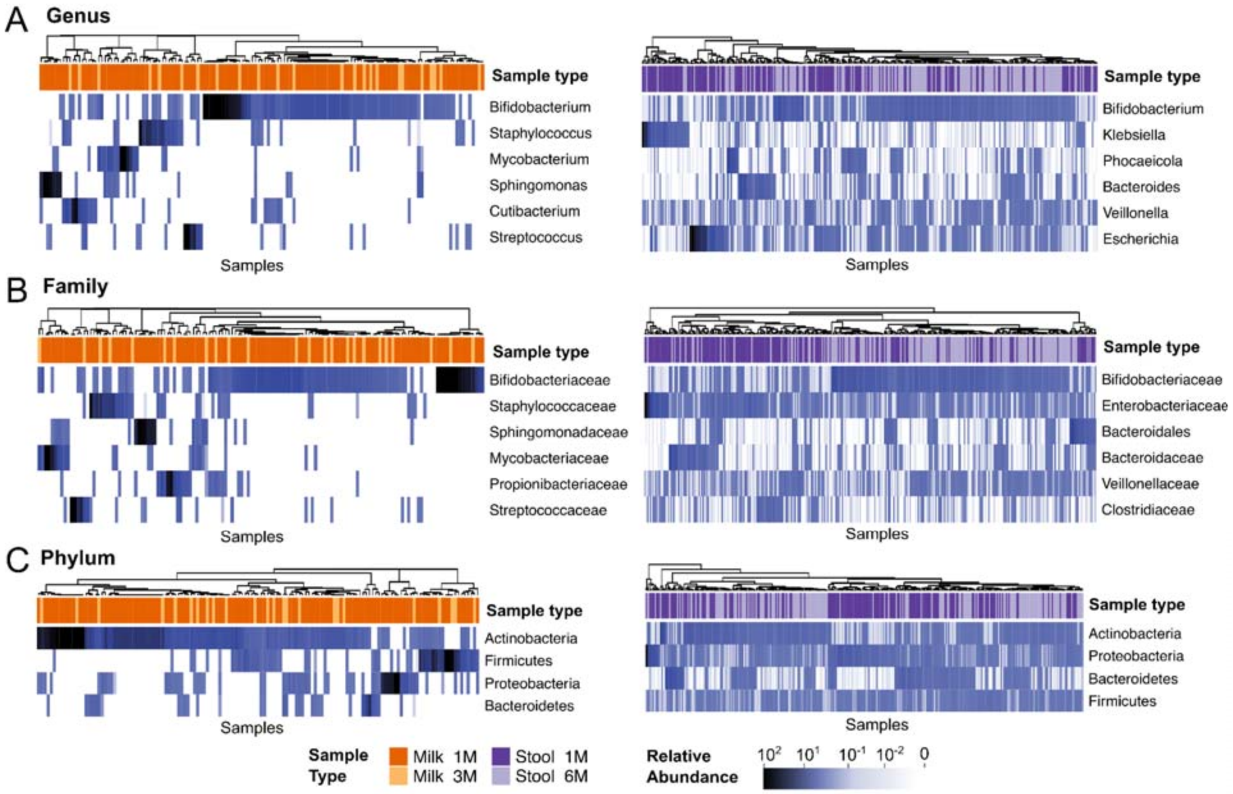

Extended Data 3. Top six genera (A) and families (B) and top four phyla (C) in milk (left) and stool samples (right).

bioRxiv preprint doi: https://doi.org/10.1101/2024.01.28.577594; this version posted January 28, 2024. The copyright holder for this preprint (which was not certified by peer review) is the author/funder, who has granted bioRxiv a license to display the preprint in perpetuity. It is made available under aCC-BY 4.0 International license.

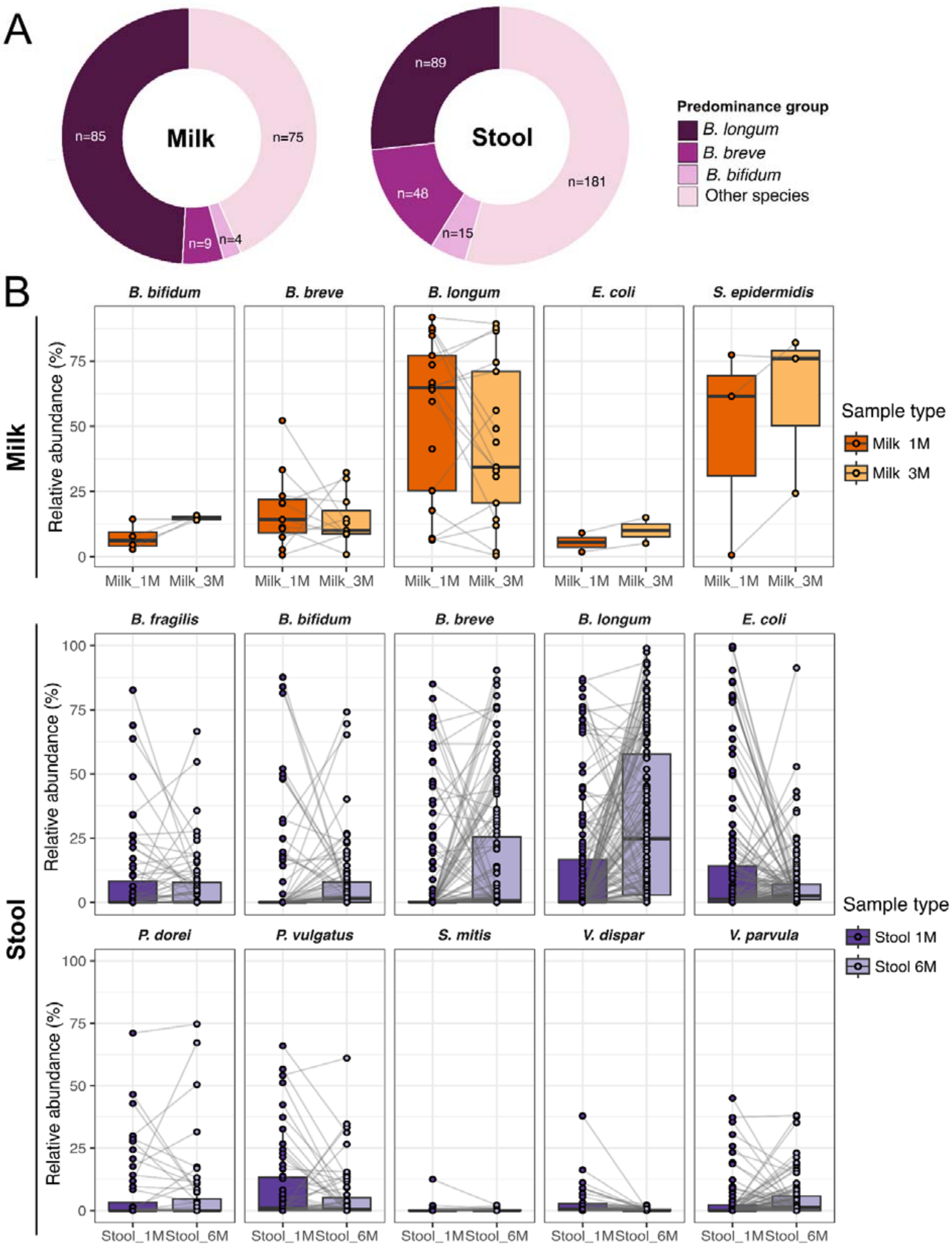

Extended Data 4. (A) Samples distributions across the four predominance groups and their associated metadata. (B) Individual species-specific relative abundance trajectories over time, for milk and infant stool samples.

bioRxiv preprint doi: https://doi.org/10.1101/2024.01.28.577594; this version posted January 28, 2024. The copyright holder for this preprint (which was not certified by peer review) is the author/funder, who has granted bioRxiv a license to display the preprint in perpetuity. It is made available under aCC-BY 4.0 International license.

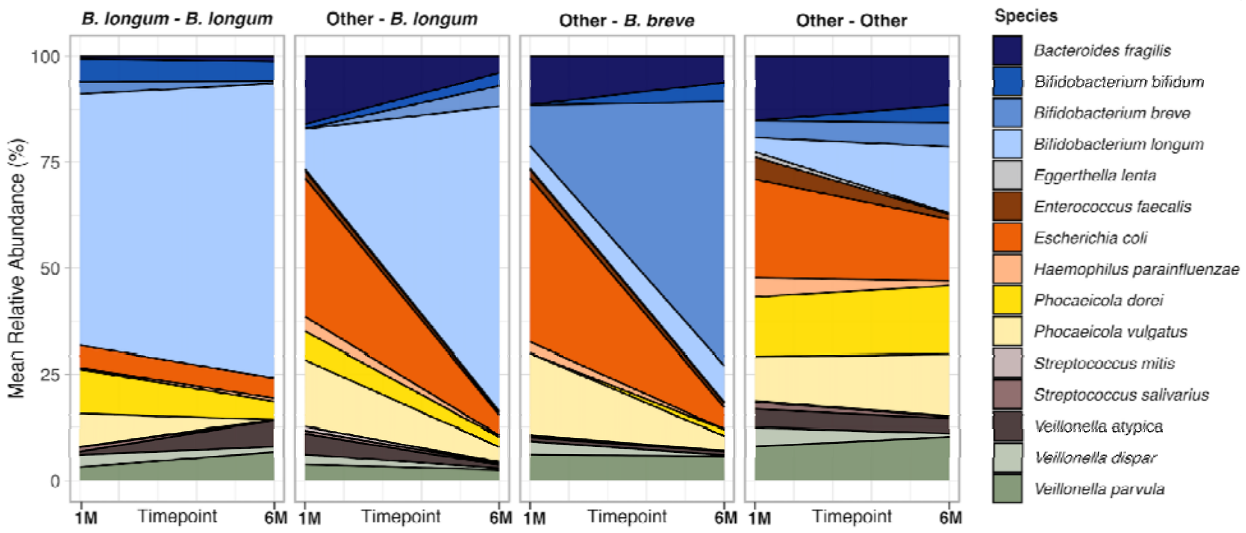

Extended Data 5. Species persistence in the infant gut microbiome over time, stratified by the type of transition between predominance groups from one to six. Only samples with both time points available and transition types with more than ten samples per type were included.

bioRxiv preprint doi: https://doi.org/10.1101/2024.01.28.577594; this version posted January 28, 2024. The copyright holder for this preprint (which was not certified by peer review) is the author/funder, who has granted bioRxiv a license to display the preprint in perpetuity. It is made available under aCC-BY 4.0 International license.

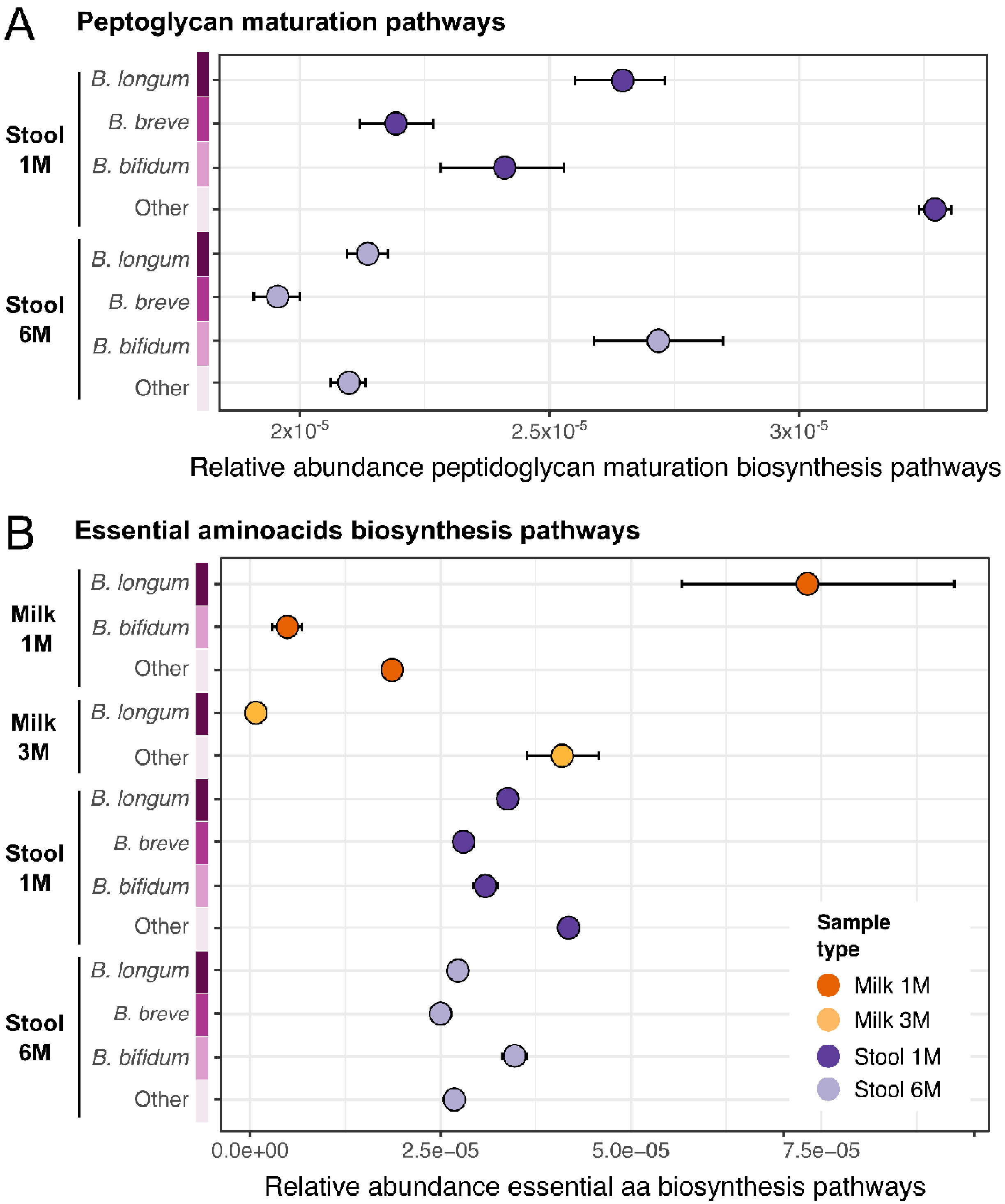

Extended Data 6. Relative abundance of pathways associated with (A) peptidoglycan maturation and (B) essential amino acids biosynthesis across sample types and (stool) predominance groups. CI at 95%, bootstrapping n=1000.

bioRxiv preprint doi: https://doi.org/10.1101/2024.01.28.577594; this version posted January 28, 2024. The copyright holder for this preprint (which was not certified by peer review) is the author/funder, who has granted bioRxiv a license to display the preprint in perpetuity. It is made available under aCC-BY 4.0 International license.

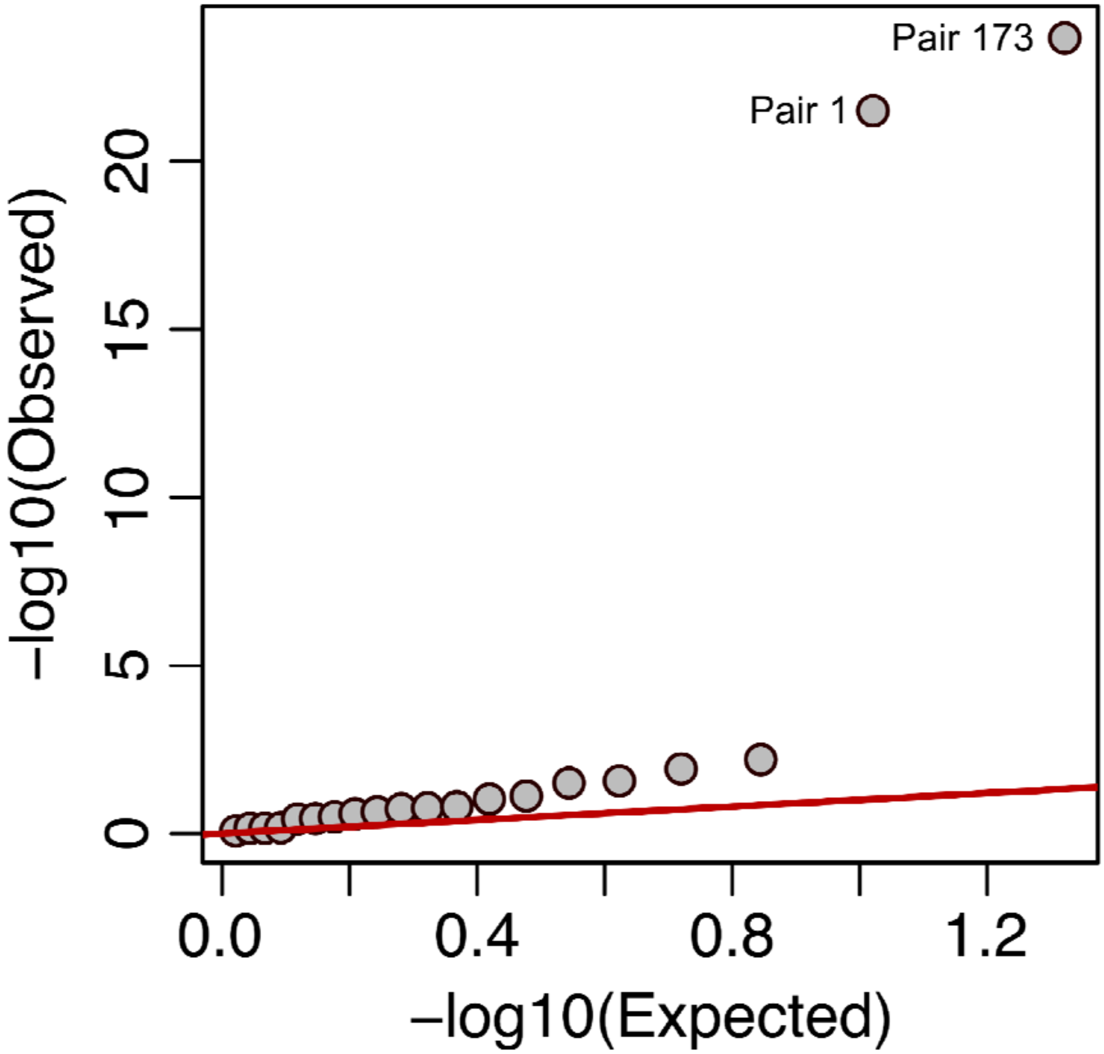

Extended Data 7. Q-Qplot of the Spearman's correlation p-values obtained comparing all metabolic pathways shared between milk and infant stools, for each mother-infant pair. Each dot represents the p-value for one mother-infant pair. Mother-infant pairs 1 and 173 are highlighted. The red line indicates a uniform distribution.

bioRxiv preprint doi: https://doi.org/10.1101/2024.01.28.577594; this version posted January 28, 2024. The copyright holder for this preprint (which was not certified by peer review) is the author/funder, who has granted bioRxiv a license to display the preprint in perpetuity. It is made available under aCC-BY 4.0 International license.

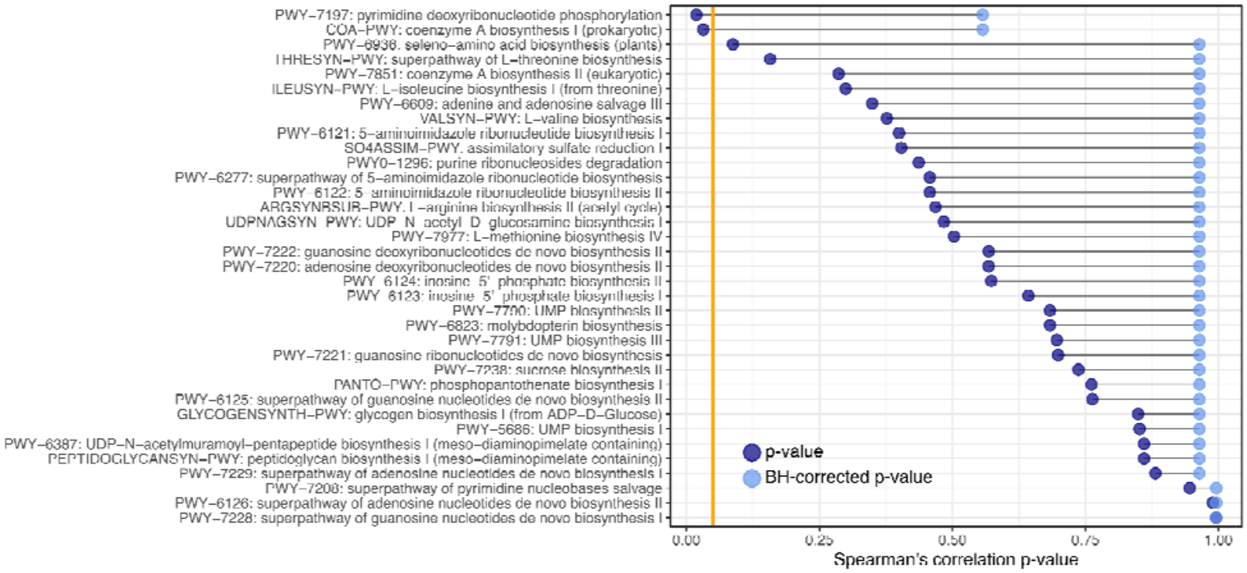

Extended Data 8. Spearman's correlation p-value for all metabolic pathways identified in both maternal milk and infant stool samples considering all mother infant pairs. The orange line identifies the significant thresholds (p=0.05). P-values were corrected for multiple testing using Benjamin Hochberg correction.

bioRxiv preprint doi: https://doi.org/10.1101/2024.01.28.577594; this version posted January 28, 2024. The copyright holder for this preprint (which was not certified by peer review) is the author/funder, who has granted bioRxiv a license to display the preprint in perpetuity. It is made available under aCC-BY 4.0 International license.

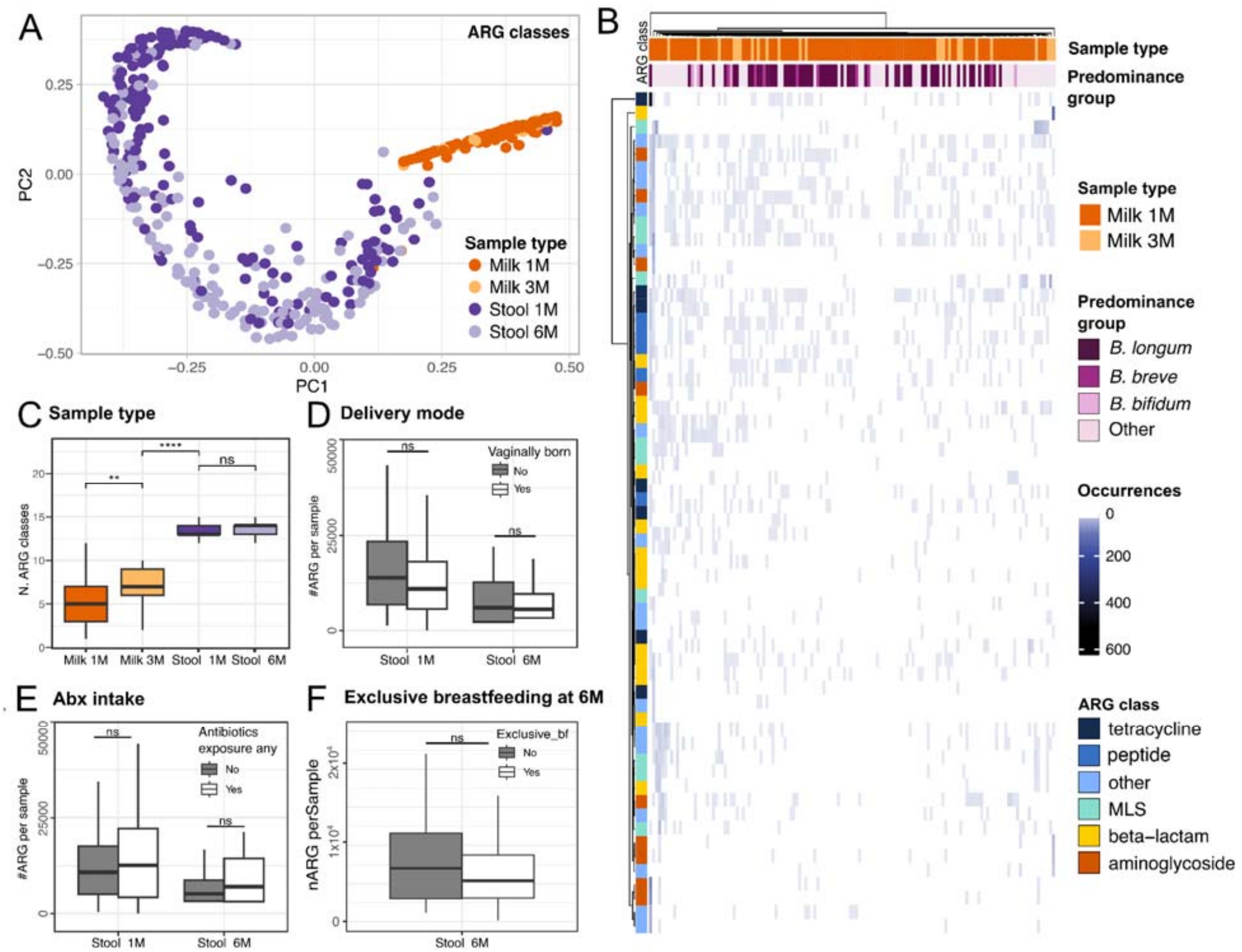

Extended Data 9. (A) PCoA of predicted ARGs classes using presence/absence information, as seen by DeepARG. (B) ARGs carriage in milk samples, divided by collection time point, most abundant species (predominance group), number of ARG genes identified and their respective ARGs class. (C) Number of distinct ARG classes identified across body sites and sampling time point. (D-F) Number of ARG genes identified in infant stool samples, divided by delivery mode, history of antibiotic intake and exclusive breastfeeding at 6 months of age, respectively. P-values calculated using t-test, **** for P ≤ 0.0001, ** p<0.01, ns for non significant.

bioRxiv preprint doi: https://doi.org/10.1101/2024.01.28.577594; this version posted January 28, 2024. The copyright holder for this preprint (which was not certified by peer review) is the author/funder, who has granted bioRxiv a license to display the preprint in perpetuity. It is made available under aCC-BY 4.0 International license.

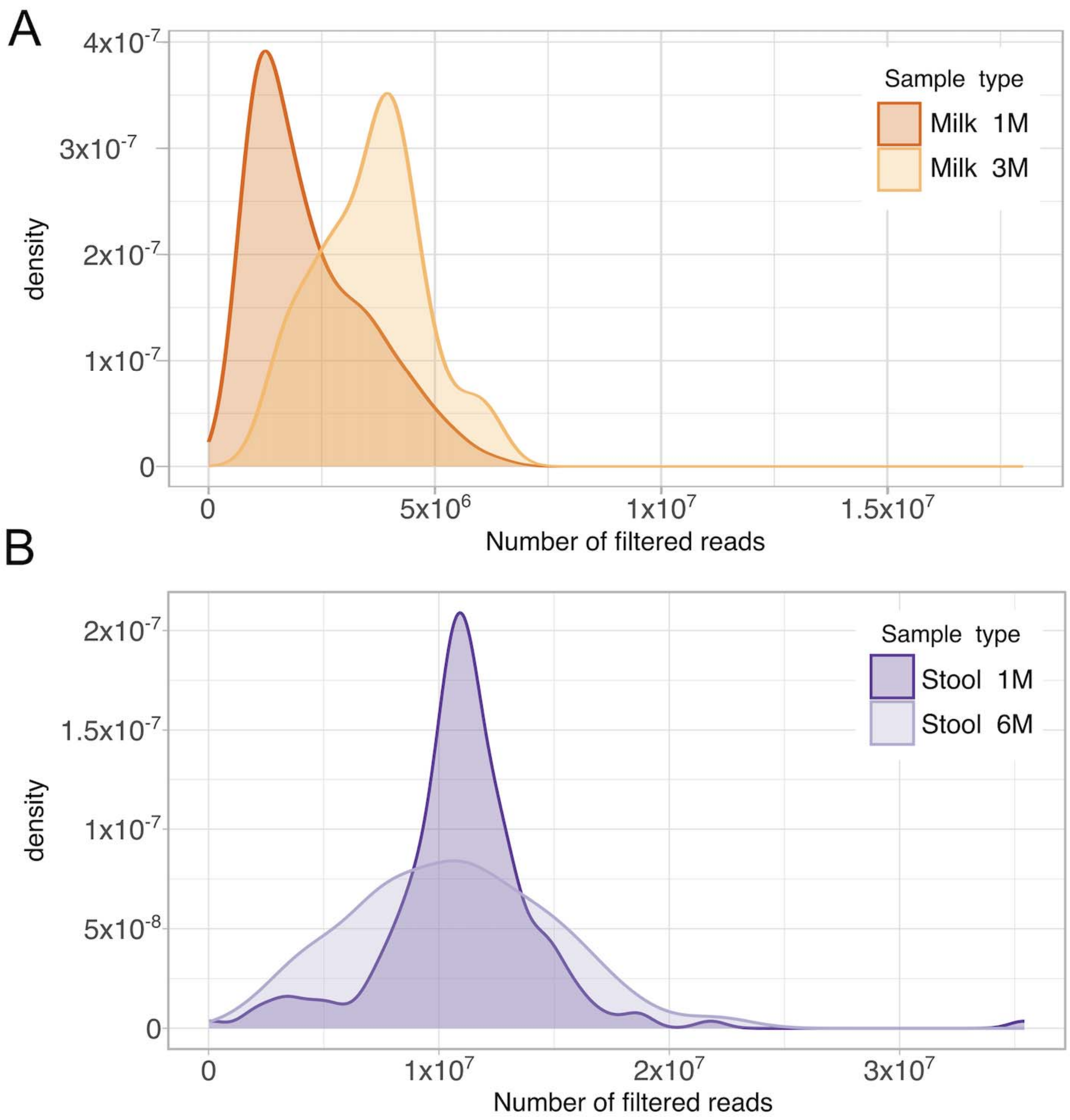

Extended Data 10. Distribution of reads after preprocessing reads divided by sample type and collection time point.

Supplementary Table 1. Samples metadata
Supplementary Table 2. Species-level taxonomic profiles as seen by MetaPhlAn4
Supplementary Table 3. Strain-level taxonomic profiles as seen by StrainPhlAn4
Supplementary Table 4. Strain persistence between stool samples at one and six months
Supplementary Table 5. HUMAnN3 functional profiles
Supplementary Table 6. DeepARG profiles
